# Widely targeted metabolome and transcriptome landscapes of *Allium fistulosum*–*A*. *cepa* chromosome addition lines revealed a flavonoid hot spot on chromosome 5A

**DOI:** 10.1038/s41598-019-39856-1

**Published:** 2019-03-05

**Authors:** Mostafa Abdelrahman, Sho Hirata, Yuji Sawada, Masami Yokota Hirai, Shusei Sato, Hideki Hirakawa, Yoko Mine, Keisuke Tanaka, Masayoshi Shigyo

**Affiliations:** 1 Botany Department, Faculty of Science, Aswan University, Aswan, 81528 Egypt; 2 Arid Land Research Center, Tottori University, Tottori-City, 680-0001 Tottori Japan; 3 Nakahara Seed Product Co., Ltd., Hakata-ku, Fukuoka-City, 812-0893 Fukuoka Japan; 4 Metabolic Systems Research Team, RIKEN Center for Sustainable Resource Science, Yokohama-City, Kanagawa 230-0045 Japan; 5 Graduate School of Life Sciences, Tohoku University, Sendai-City, 980-8577 Miyagi Japan; 6 Facility for Genome Informatics, Kazusa DNA Research Institute, Kisarazu-City, 292-0818 Chiba Japan; 7 Faculty of Agriculture, Tokyo University of Agriculture, Atsugi-City, 243-0034 Kanagawa Japan; 8 NODAI Genome Research Center, Tokyo University of Agriculture, Setagaya-ku, 156-8502 Tokyo Japan; 9 College of Agriculture, Graduate School of Sciences and Technology for Innovation, Yamaguchi University, Yamaguchi-City, 753-8515 Yamaguchi Japan

## Abstract

Here, we report a comprehensive analysis of the widely targeted metabolome and transcriptome profiles of *Allium fistulosum* L. (FF) with the single extra chromosome of shallot [*A*. *cepa* L. Aggregatum group (AA)] to clarify the novel gene functions in flavonoid biosynthesis. An exhaustive metabolome analysis was performed using the selected reaction monitoring mode of liquid chromatography–tandem quadrupole mass spectrometry, revealing a specific accumulation of quercetin, anthocyanin and flavone glucosides in AA and FF5A. The addition of chromosome 5A from the shallot to *A*. *fistulosum* induced flavonoid accumulation in the recipient species, which was associated with the upregulation of several genes including the *dihydroflavonol 4-reductase*, *chalcone synthase*, *flavanone 3-hydroxylase*, *UDP-glucose flavonoid-3-O-glucosyltransferase*, *anthocyanin 5-aromatic acyltransferase-like*, *pleiotropic drug resistance-like ATP binding cassette transporter*, and *MYB14* transcriptional factor. Additionally, an open access *Allium* Transcript Database (*Allium* TDB, http://alliumtdb.kazusa.or.jp) was generated by using RNA-Seq data from different genetic stocks including the *A*. *fistulosum–A*. *cepa* monosomic addition lines. The functional genomic approach presented here provides an innovative means of targeting the gene responsible for flavonoid biosynthesis in *A*. *cepa*. The understanding of flavonoid compounds and biosynthesis-related genes would facilitate the development of noble *Allium* varieties with unique chemical constituents and, subsequently, improved plant stress tolerance and human health benefits.

## Introduction

*Allium* is an enormous genus (850 species) that compromises several economically important crops, including the bulb onion (*Allium cepa*), shallot (*A*. *cepa* Aggregatum group), Japanese bunching onion (*A*. *fistulosum*), garlic (*A*. *sativum*), chive (*A*. *schoenoprasum*), Chinese chive (*A*. *tuberosum*), and leek (*A*. *ampeloprasum*). Onions and garlic are some of the earliest domesticated horticultural crops that have been broadly cultivated and prized for their nutritional, medicinal, and culinary properties^1–3^. The total dry onion production in 2016 was ~93.1 million tons (MTs), of which 61.0 MT was produced in Asia, 12.41 in Africa, 10.11 in the Americas, and 9.36 in Europe (www.fao.org, accessed in 2018). *Allium* species are located across the northern hemisphere, mainly in the semiarid regions of North America, Europe, North Africa, and Asia, a region with diverse ecological stretches that led to the development of an amazing number of *Allium* species with different physiological and morphological traits^3–6^. However, the unbalanced selections by breeders and farmers have resulted in the loss of many useful agronomic traits in *Allium* crops^1,7^. Novel alleles with desirable attributes can be introduced by crossing *Allium* crops with wild or landrace progenitors to develop new *Allium* varieties.

In Southeast Asian countries, shallots (2*n* = 16), which are genetically closer to the bulb onion, have been recognized as a potential genetic reserve for *Allium* crop improvement because of their adaptability to a wide range of environmental stresses^3,8,9^. The integrated transcriptome and phytochemical analyses of monosomic addition lines (MALs, 2*n* = 17) of the Japanese bunching onion (FF, 2*n* = 16) with a single extra chromosome from the shallot (AA) enabled us to identify several transcripts on the FF genome with extra chromosome 2A from the shallot (FF2A), that involved in Alliospiroside A biosynthesis, a major saponin compound contributed to shallot resistance against the *Fusarium* pathogen^3^. Our early studies^10,11^ also showed a considerable accumulation of carbohydrates and amino acids in several MALs during the winter and summer seasons, respectively. These reports gave insight into the significant effect of shallot extra chromosomes on the metabolic profile of *A*. *fistulosum* despite the fact that the causal genes regulating this process remain elusive.

Flavonoids are widely distributed in several plant species with diverse biological functions, including protection against abiotic/biotic stresses, male fertility, plant signaling, auxin transport, and color development to attract pollinators, as well as diverse human health–promoting properties^12–14^. Bulb onions and shallots are known to contain a large amount of flavonols, especially quercetin, quercetin-4′-glucoside, and quercetin-3,4′-diglucoside, as well as anthocyanins in comparison with *A*. *fistulosum*^9,15,16^. These flavonoid compounds are usually found at high concentrations in the outer skin of onion and shallot bulbs, where they impart yellow-red color development^17^. *S*-alk(en)yl-L-cysteine sulfoxides (ACSOs) are also one of the most characteristic ingredients in *Allium* species, playing a key role in both chemotaxonomic values and biological activities^18^. (+)-*S*-methyl-L-cysteine sulfoxide (Methiin, MeCSO), which is associated with a hot pungent taste and strong sulfur odor, is ubiquitous in the genus *Allium*, while (+)-*S*-(2-Propenyl)-L-cysteine sulfoxide (Alliin, AlCSO) and (+)-*S*-(trans-1-Propenyl)-L-cysteine sulfoxide (Isoalliin, PrenCSO) are characteristic of garlic and onions, respectively^1,19^.

The plant metabolome can offer a snapshot of the biochemical and physiological status of a plant cell at a given developmental stage under a particular set of environmental conditions, and thus it is closely related to the plant phenotypes^20–22^. In addition, plant metabolomic explains many of biological changes caused by genetic or environmental perturbations; thus it can be used to evaluate the effect of a foreign gene(s) and understand the relationships between gene function and metabolic phenotypes^23^. With the recent development of next-generation sequencing (NGS) technologies to outline gene expression profiles in the context of plant–environment interaction, integrated metabolome and transcriptome analyses are regarded as a powerful method to understand the molecular machinery underlying various biological process^9,24^. In this context, we performed integrated transcriptome and widely targeted metabolome analyses of *A*. *fistulosum* (FF), the shallot (AA), and MALs (FF1A, FF2A, FF3A, FF4A, FF5A, FF6A, FF7A, and FF8A) to investigate the effect of adding a single chromosome from the shallot to the *A*. *fistulosum* metabolome with a particular focus on flavonoids as a metabolic phenotype characteristic for the shallot.

## Results

### Flavonoids and ACSO profiling of FF, AA, and MALs

**Figure 1:**
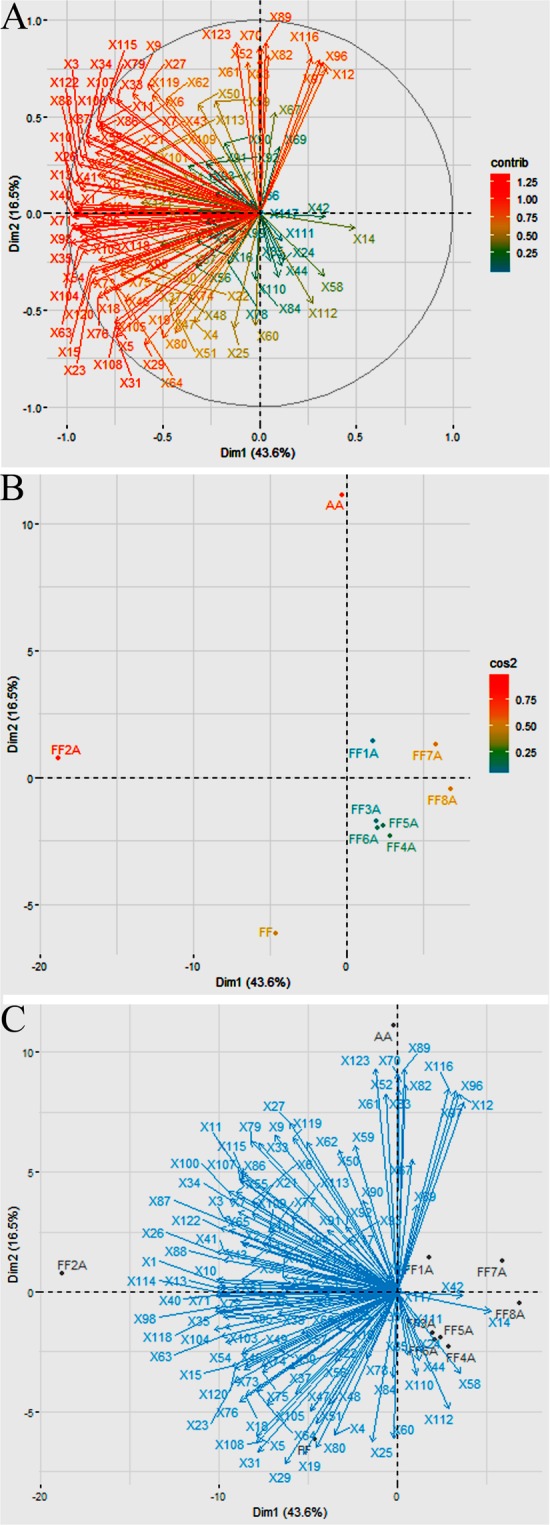
Principal component analysis (PCA) of the normalized metabolite intensities in *Allium fistulosum* (FF), the *A*. *cepa* Aggregatum group (AA), and monosomic addition lines (MALs = FF1A, FF2A, FF3A, FF4A, FF5A, FF6A, FF7A, and FF8A). (**A**) PCA loading plot of PC1 against PC2. Each metabolite is represented by a single dot. (**B**) PCA plot showing separation by genotype. The accumulation of variance percentage is indicated in each PC. (**C**) PCA biplot of metabolite and genotype segregation.

In this study, we profiled a widely targeted metabolome and transcriptome profile in bulb tissues of the Japanese bunching onion (FF), shallot (AA), and MALs (FF1A, FF2A, FF3A, FF4A, FF5A, FF6A, FF7A, and FF8A). We obtained 499 metabolite intensities via selected reaction monitoring (SRM) of liquid chromatography–tandem quadrupole mass spectrometry (LC-QqQ-MS) analysis (Supplementary Table S1). Seventy-nine flavonoids and eight ACSO secondary metabolites were detected in FF, AA, and MALs by LC-QqQ-MS (Supplementary Table S6). The metabolite data matrix was further filtered by removing the metabolites with signal-to-noise ratio (S/N) < 5 and relative standard deviation (RSD) > 0.30, leaving 123 metabolites for further analysis (Supplementary Table S5). All of the 123 metabolites were subjected to enrichment pathway analysis using MetaboAnalyst 4.0 (Supplementary Fig. S1 and Supplementary Table S7). Out of 123 metabolites, 113 were found in the MetaboAnalyst server database for *Arabidopsis*, representing several metabolic pathways (Supplementary Fig. S1 and Supplementary Table S7). Next, all of the 123 metabolites were subjected to principal component analysis (PCA) to gain further insight into the contribution of various metabolites in FF, AA, and MALs (Fig. 1). All of the 123 metabolites were loaded into two major principal components (PC1 and PC2), explaining 60.10% of the variance (Fig. 1A). FF, AA, and eight MALs were contributed to PC1 and explained a large proportion of the variance (43.60%), while the lower proportion of variance (16.50%) was explained by PC2 (Fig. 1B). The PCA plot divides the genotypes into three groups: FF and FF2A were clustered in one group, AA in a second group, and FF1A, FF3A, FF4A, FF5A, FF6A, FF7A, and FF8A in a third group (Fig. 1B). In the PCA biplot, sucrose, quercetin 3-galactoside, quercetin-4′-glucoside, petunidin 3-O-glucoside, quercetin-3,4′-diglucoside, lactulose, melibiose, and 1F-beta-D-fructosylsucrose (X12, X52, X70, X82, X83, X96, X97, and X116, respectively) were closely associated with the AA genotype (Fig. 1C). Similarly, 1-amino-1-cyclopentanecarboxylic acid, adenosine, pyridoxamine, 3,4-dihydroxy-L-phenylalanine, threo-3-methylaspartate, guanosine, 5-oxo-L-proline, sn-glycero-3-phosphocholine, pipecolate, methionine sulfoxide, and lysine (X1, X5, X13, X19, X26, X29, X40, X64, X80, X88, and X114, respectively) were highly associated with the FF and FF2A genotypes, whereas 3-methyladenine, arabinose, maltotriose, O-phospho-L-serine, galactitol, xylulose, 2-oxoglutarate, carnitine, and gamma-Glu-PRENCS (X14, X24, X42, X44, X58, X110, X111, X112, and X117, respectively) were closely associated with different MALs (Fig. 1C).

**Figure 2:**
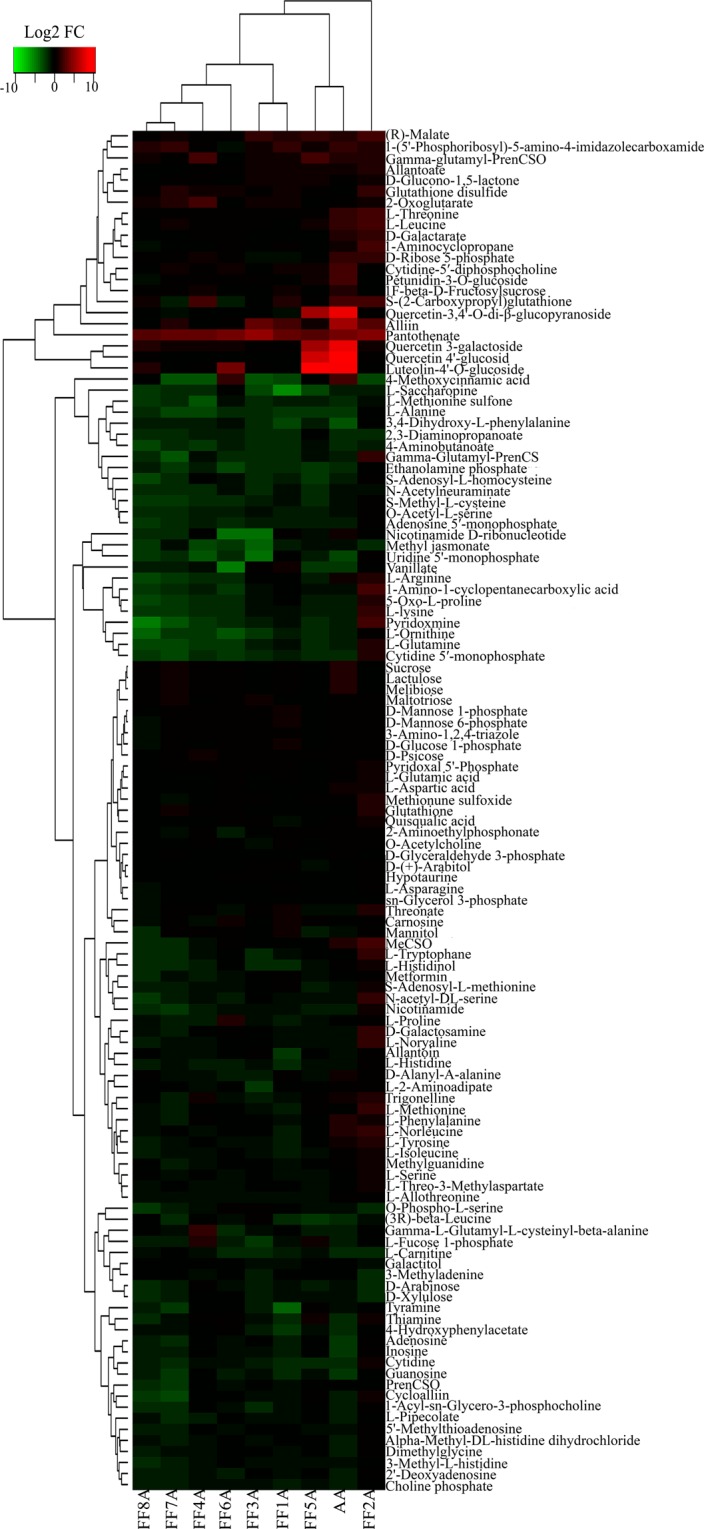
Heatmap hierarchical clustering showing metabolite fold change in the *Allium cepa* Aggregatum group (AA) and monosomic addition lines (MALs = FF1A, FF2A, FF3A, FF4A, FF5A, FF6A, FF7A, and FF8A) in comparison with *A*. *fistulosum* (FF) as a control. The color scale for hierarchical clustering is labeled.

**Figure 3:**
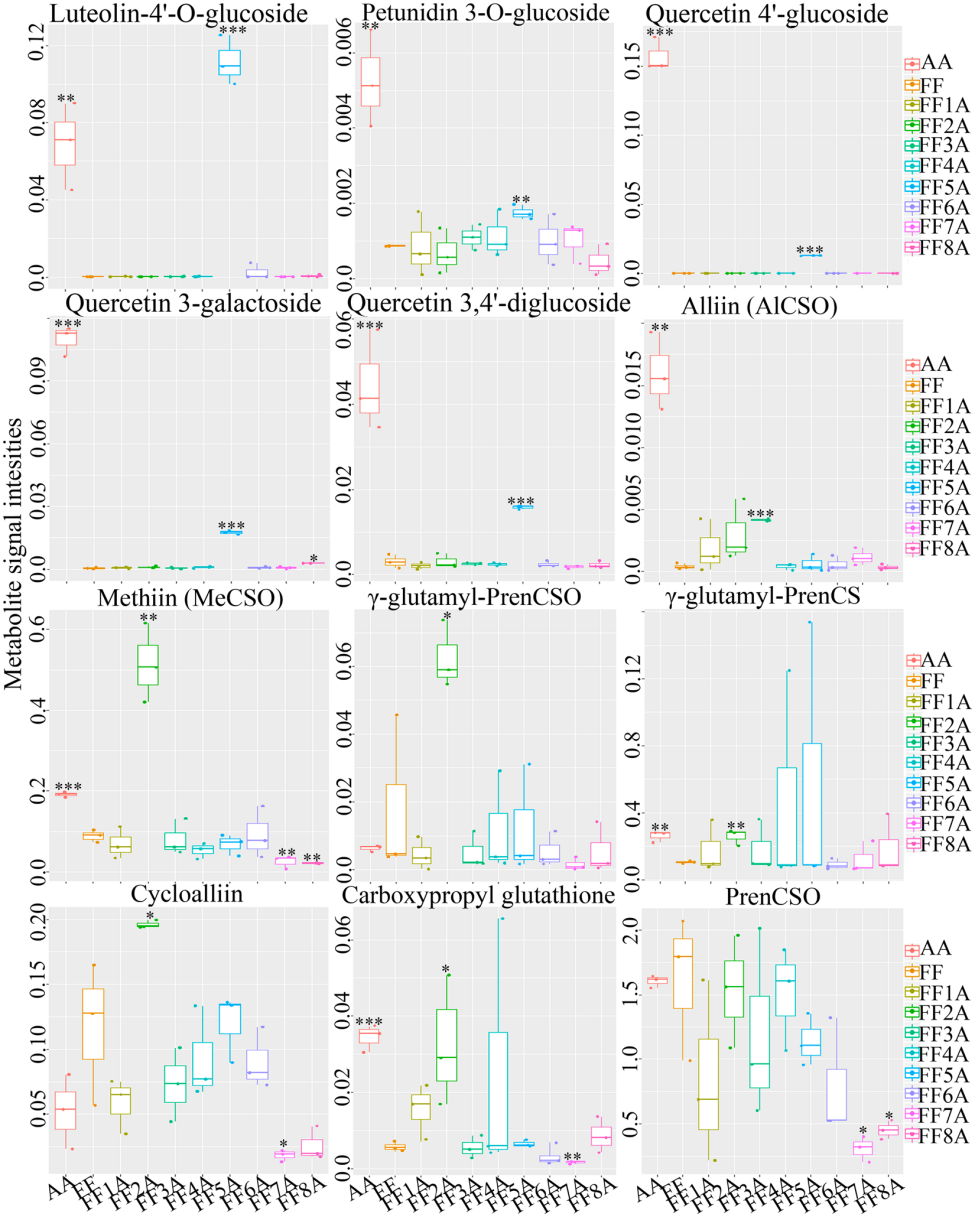
Box plot for the temporal variability of key flavonoid and cysteine sulfoxide (CSO) compound signal intensities in *Allium fistulosum* (FF), the *A*. *cepa* Aggregatum group (AA), and monosomic addition lines (MALs = FF1A, FF2A, FF3A, FF4A, FF5A, FF6A, FF7A, and FF8A). The box boundary represents the 25th percentile, median, and 75th percentile. The error bars above and below the box indicate the 90th percentile. Values represent three independent biological replicates (n = 3), and significance levels are given as *(*P* < 0.05), **(*P* < 0.01), and ***(*P* < 0.001), according to Student’s *t*-test.

**Figure 4:**
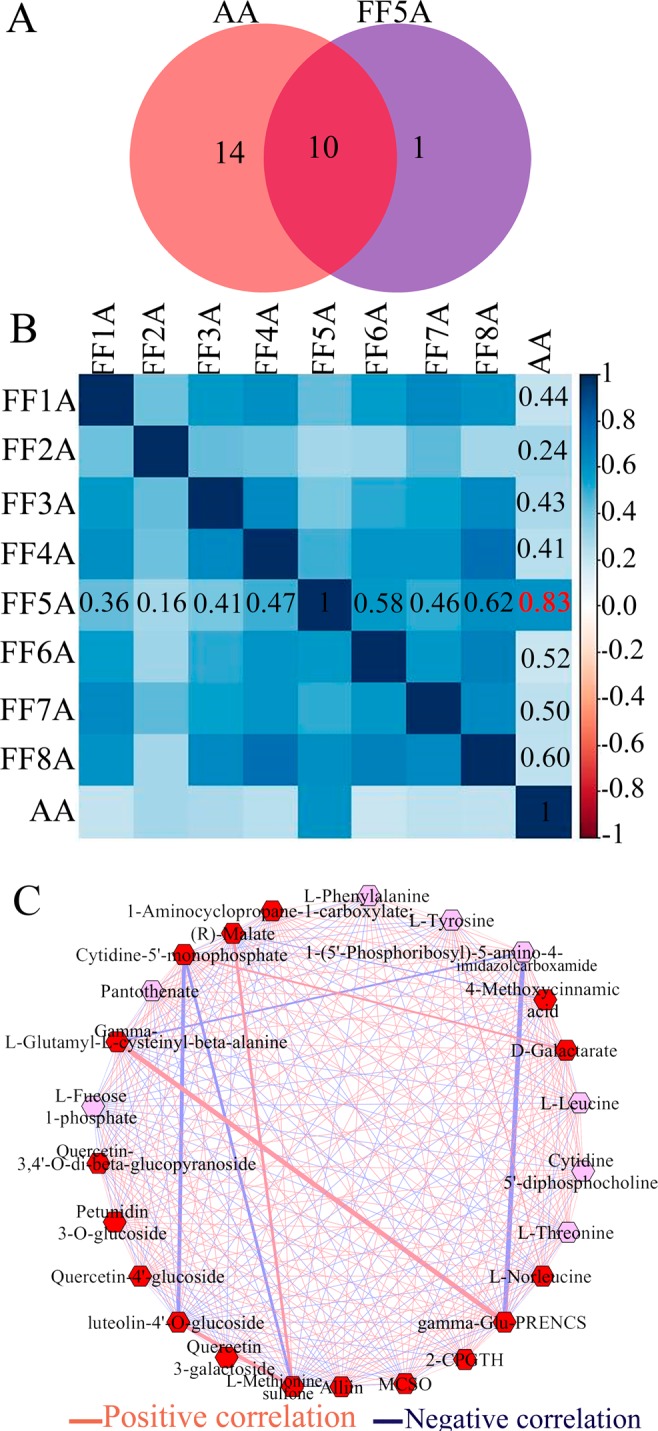
Venn diagram, Pearson correlation coefficient, and metabolite network of the significantly associated metabolites in the *A*. *cepa* Aggregatum group (AA) and monosomic addition lines (MALs = FF1A, FF2A, FF3A, FF4A, FF5A, FF6A, FF7A, and FF8A) relative to *A*. *fistulosum* (FF). (**A**) Venn diagram of the specific metabolites and those overlapping with either AA or FF5A. (**B**) Pearson correlation coefficient based on the metabolite fold change of AA and MALs using FF as a control. (**C**) The metabolite network was generated based on the Pearson correlation coefficient using Correlation Calculator 1.0.1, and the network was plotted using Cytoscape 3.3.0.

To obtain a better metabolite segregation within AA and MALs, we performed a log2 transformation of metabolite fold changes using the FF genotype as a control and the resulting data matrix was analyzed using heatmap hierarchical clustering (Fig. 2). The heatmap showed a clear segregation between AA and MALs. For example, the FF2A genotype was separated as one group, similar to the PCA result, whereas the FF5A and AA genotypes were clustered together (Fig. 2). Several metabolites were induced in the FF2A genotype, especially amino acid and ACSOs (Figs 2, 3 and Table 1). However, all five flavonoid compounds, luteolin-4′-O-glucoside, quercetin-4′-glucoside, quercetin 3-galactoside, quercetin-3,4′-O-di-beta-glucopyranoside/quercetin-3,4′-diglucoside, and petunidin 3-O-glucoside, were exclusively detected in the FF5A and AA genotypes when compared with FF and other MALs (Figs 2, 3 and Table 1). Among all metabolites, luteolin-4′-O-glucoside, quercetin-4′-glucoside, and quercetin 3-galactoside were the most highly accumulated ones, with a 382.45-, 399.28-, and 234.65-fold increase in AA and a 621.05-, 118.86-, and 37.45-fold increase in FF5A relative to FF (Table 1). In total, 24 metabolites were significantly (*P* < 0.05) changed in AA, and 11 metabolites were significantly (*P* < 0.05) changed in FF5A in comparison with FF (Fig. 4A and Table 1). Of these, seven metabolites exhibited similar increasing trend in both AA and FF5A, while three metabolites displayed similar decreasing tendency in both AA and FF5A in comparison with FF (Fig. 4A, Table 1). The ACSO metabolites, including, gamma-glutamyl-PrenCS, *S*-2-carboxypropyl glutathione (2-CPGTH) and MeCSO, were significantly (*P* < 0.05) accumulated in AA and FF2A genotypes, AlCSO in AA and FF3A genotypes, and gamma-glutamyl-PrenCSO and cycloalliin in FF2A genotype in comparison with FF (Fig. 3, Table 1). On the other hand, MeCSO, and PrenCSO were significantly (*P* < 0.05) decreased in both FF7A and FF8A genotypes relative to FF (Fig. 3 and Table 1). A Pearson correlation coefficient (Pcc) was carried out using a data matrix (log2 fold change) to identify the relationship between AA and MALs based on their metabolite level (Fig. 4B). Our result indicated a highly significant positive correlation between AA-FF5A (*r* = 0.83), FF5A-FF8A (*r* = 0.62), and AA-FF8A (*r* = 0.60), whereas AA-FF2A genotypes exhibited a weak positive correlation (*r* = 0.24). In addition, a metabolite network based on the Pcc demonstrated a highly positive correlation between gamma-L-glutamyl-L-cysteinyl-beta-alanine and gamma-glutamyl-PrenCS (*r* = 0.87), followed by luteolin-4′-O-glucoside and methionine sulfone (*r* = 0.71) (Fig. 4C). A negative correlation was observed between gamma-glutamyl-PrenCS and 1-(5′-phosphoribosyl)-5-amino-4-imidazolecarboxamide (*r* = −0.70) and between luteolin-4′-O-glucoside and cytidine-5′-monophosphate (*r *= −0.55) (Fig. 4C).

**Table 1:** Metabolite fold changes in *Allium cepa* Aggregatum group (AA) and *A*. *fistulosum* (FF) with extra chromosome from *A. cepa* (1A-8A).

Metabolites	Genotypes								
	FF1A	FF2A	FF3A	FF4A	FF5A	FF6A	FF7A	FF8A	AA
	Fold change								
1-Aminocyclopropane-1-carboxylate	1.08	4.89^**^	0.84	0.77	0.81	1.02	0.72	0.64	2.03^*^
1-Amino-1-cyclopentanecarboxylic acid	0.92	6.10^***^	0.54	0.34	0.51	0.21	0.23	0.19	0.51
2-Oxoglutarate	1.77	1.84	1.61	4.82^*^	0.78	0.97	2.15	1.74	1.00
L-Methionine	0.41	3.40^**^	0.64	0.77	1.11	0.70	0.37	0.49	0.88
L-Phenylalanine	0.62	2.03^**^	0.87	1.00	0.77	0.70	0.46^*^	0.48^*^	2.29^*^
L-Tryptophan	0.49	3.02^*^	0.29	0.56	0.71	0.97	0.28	0.29	0.87
L-Tyrosine	0.40	2.56^*^	0.61	0.76	0.91	0.63	0.54	0.40	2.04^*^
L-Isoleucine	0.33	1.98^*^	0.64	0.60	0.49	0.90	0.59	0.46	1.29
L-Leucine	0.76	5.28^**^	1.16	1.17	1.52	1.34	1.53	0.93	3.12^*^
L-Norleucine	0.41	3.10^*^	0.75	0.57	0.91	0.87	0.85	0.54	2.24^*^
L-Threonine	1.06	5.63^**^	1.37	1.20	1.27	1.26	1.14	1.24	3.30^**^
(R)-Malate	2.69^*^	4.97^**^	3.36^***^	1.37	3.04^*^	1.36	1.55	1.03	2.42^*^
L-Norvaline	0.65	3.54^**^	0.83	0.52	0.55	0.85	0.66	0.40	0.65
L-Lysine	0.69	4.02^*^	0.54	0.26	0.35	0.24	0.16^*^	0.19^*^	0.43
Histamine	0.31	1.38	0.54	0.46^*^	0.54	0.36	0.52	0.42	0.54
D-Xylulose	0.58	0.26^*^	0.35	0.97	0.50	0.71	0.40	0.29^*^	0.58
D-Galactarate	0.95	2.97^*^	0.95	1.03	0.86	1.06	0.88	0.70	2.57^*^
D-Galactosamine	0.64	3.00^*^	0.84	0.79	0.53	1.10	0.46	0.62	0.55
sn-glycero-3-Phosphocholine	0.49	1.00	0.28	0.80	0.77	0.54	0.24^*^	0.27	0.36
Pyridoxamine	0.62	4.99^**^	0.46	0.20	0.30	0.30	0.08	0.03	0.45
N-acetyl-DL-serine	0.48	3.56^*^	0.71	0.63	0.60	0.48	0.44	0.17	0.58
1-(5′-Phosphoribosyl)-5-amino-4-imidazolecarboxamide	3.10	2.84	1.73	1.28	2.05	0.67	3.65^**^	2.09	3.72^*^
4-Methoxycinnamic acid	0.11	0.12	0.11	0.10	0.72	3.80	0.10	0.80	4.52^*^
Luteolin-4′-O-glucoside	0.94	0.90	1.33	1.27	621.05^***^	14.30	0.74	2.90	382.45^**^
Quercetin-4′-glucoside	0.95	1.03	0.92	0.87	118.86^***^	0.92	0.91	1.37	1399.28^***^
Quercetin 3-galactoside	1.21	1.86	1.11	1.83	37.45^***^	1.51	1.46	2.54^*^	234.65^***^
Petunidin 3-O-glucoside	0.97	0.79	1.26	1.29	2.01^**^	1.14	1.17	0.52	6.05^**^
Quercetin-3,4′-O-di-beta-glucopyranoside	0.68	1.00	0.83	0.81	5.19^***^	0.81	0.58	0.75	14.60^**^
L-Fucose 1-phosphate	0.58	0.70	0.22^**^	2.09^*^	1.64^*^	0.38^**^	0.35^*^	0.40^**^	0.46
Cytidine-5′-monophosphate	0.38	2.71^*^	0.25	0.26	0.26^*^	0.19^*^	0.15^*^	0.19^*^	0.30^*^
Uridine 5′-monophosphate	0.69	1.28	0.05^*^	0.10^*^	0.46	0.31	0.25	0.18^*^	0.15^*^
Pantothenate	11.78^***^	18.73^**^	29.52^*^	10.21^*^	7.87^**^	15.29^*^	9.77	7.65	18.12^**^
L-Methionine sulfone	0.46^*^	0.64	0.27^**^	0.10^**^	0.46^**^	0.69	0.27^**^	0.22^**^	0.30^*^
Methionine sulfoxide	0.94	2.66^*^	1.18	0.90	1.11	1.08	0.68	0.91	1.22
gamma-L-glutamyl-L-cysteinyl-beta-alanine	0.89	0.77	0.60	3.28^*^	0.46^*^	0.25^*^	0.63	0.71	0.46^*^
gamma-glutamyl-PrenCSO	0.24	3.45^*^	0.28	0.64	0.68	0.29	0.08	0.30	0.34
gamma-glutamyl-PrenNCS	1.69	2.4^**^	1.74	4.49	5.43	0.87	1.17	1.79	2.49^**^
2-CPGTH	2.76	5.74^*^	0.96	4.36	1.14	0.47	0.38^**^	1.50	5.95^***^
PrenCSO	0.51	0.94	0.73	0.93	0.70	0.48	0.18^*^	0.27^*^	0.99
Methiin (MeCSO)	0.77	5.77^**^	0.90	0.59	0.76	1.03	0.28^**^	0.24^**^	2.14^***^
Alliin (AlCSO)	4.65	7.46	10.37^***^	0.99	1.51	1.40	2.86	0.85	39.83^**^
Cycloalliin	0.50	1.68^*^	0.62	0.79	1.02	0.77	0.15^*^	0.22	0.45

Significance levels are given as: *(*P* < 0.05), **(*P* < 0.01) and ***(*P* < 0.001) according to student’s *t*-test. Fold change calculated based on metabolite signal intensities in AA and MALs versus FF as control.

### Transcriptome profile of flavonoid biosynthesis and glycosylation genes in FF, AA, and MALs

**Figure 5:**
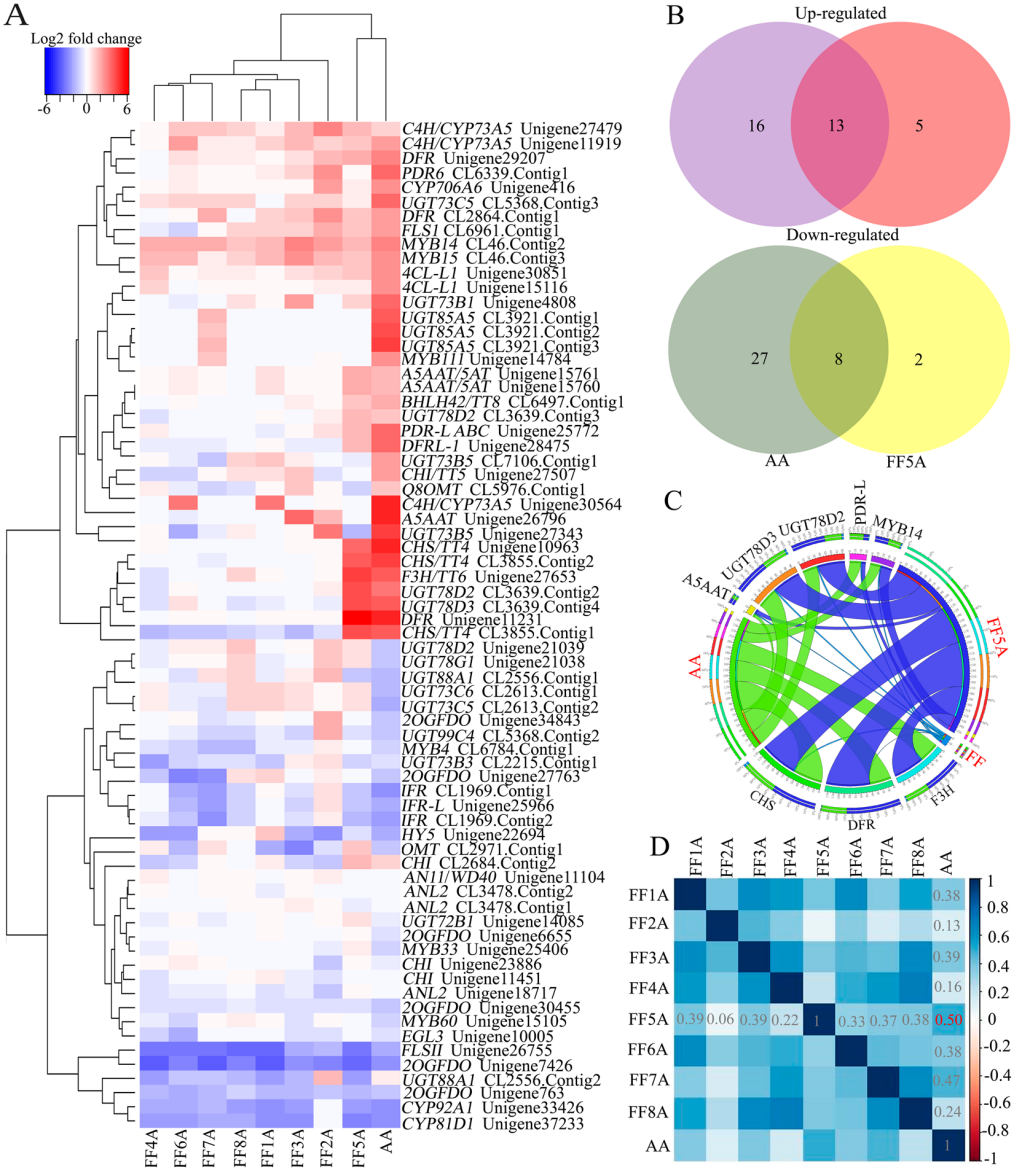
Differential gene expression level of flavonoid-related genes in the *Allium cepa* Aggregatum group (AA) and monosomic addition lines (MALs = FF1A, FF2A, FF3A, FF4A, FF5A, FF6A, FF7A, and FF8A) relative to *A*. *fistulosum* (FF) as a control. (**A**) Heatmap hierarchical clustering showing gene expression fold changes in AA and MALs. (**B**) Venn diagram of up- and downregulated flavonoid-related genes in AA and FF5A. (**C**) Circos plot of differentially upregulated genes in AA and FF5A relative to FF. Read per kilobase per million mapped reads (RPKM) were used to generate the Circos plot. (**D**) Pearson correlation coefficient between AA and MALs based on the flavonoid gene expression fold change. *Dihydroflavonol 4-reductase*, *DFR*; *DFR-like*, *DFR-L*; *chalcone synthase*, *CHS*; *flavanone 3-hydroxylase*, F3H; *UDP-glucose flavonoid-3-O-glucosyltransferase*, *UGT78D2* and *UGT73D3*; *anthocyanin 5-aromatic acyltransferase-like*, *A5AAT-L*; *pleiotropic drug resistance-like ATP binding cassette transporter*, *PDR-L ABC*.

To identify the gene(s) attributed to the high accumulation of flavonoids in the FF5A and AA genotypes in comparison with FF, we blast searched the flavonoid-related genes in our *Allium* TDB using the flavonoid pathway from the Kyoto Encyclopedia of Genes and Genomes (KEGG; http://www.genome.jp/kegg/) and in published literature. We detected 159 unigenes encoded by the flavonoid regulation-, glycosylation-, and acylation-related genes in our *Allium* TDB (Supplementary Table S8). Differential gene expression (DEG) analysis was carried out by using a pairwise comparison between FF5A and AA, with FF as a control (Supplementary Table S9). Sixty-four transcripts were differentially expressed (FDR < 0.05) in AA relative to FF (Fig. 5A and Supplementary Table S9). Among these, 29 transcripts were upregulated and 35 were downregulated in the AA genotype in comparison with FF (Fig. 5B). Likewise, 28 transcripts were differentially expressed (FDR < 0.05) in FF5A in comparison with FF, among which 18 transcripts were upregulated and ten were downregulated (Fig. 5B). Thirteen transcripts encoded by the dihydroflavonol 4-reductase (DFR/TT3; Unigene11231), DFR-like (DFR-L; Unigene28475), chalcone synthase (CHS/TT4; Unigene10963, CL3855.Contig1, and CL3855.Contig2), flavanone 3-hydroxylase (F3H/TT6; Unigene27653), UDP-glucose flavonoid-3-O-glucosyltransferase [UGT78D2 (CL3639.Contig2 and CL3639.Contig3) and UGT73D3 (CL3639.Contig4)], anthocyanin 5-aromatic acyltransferase-like (A5AAT-L/5AT; Unigene15761), pleiotropic drug resistance-like ATP binding cassette transporter (PDR-L ABC; CL6497.Contig1), and MYB14 (CL46.Contig2) genes were upregulated in both AA and FF5A in comparison with FF (Figs. 5A,C). Whereas, eight transcripts encoded by the UGT73B3 (CL2215.Contig1), flavone synthase II (FLSII; Unigene26755), NmrA-like negative transcriptional regulator (Unigene25966 and CL1969.Contig1), NAD(P)-binding Rossmann-fold superfamily protein (CL1969.Contig2), 2-oxoglutarate (2OG) and Fe(II)-dependent oxygenase (2OGFDO; Unigene7426), CYP81D1/F3H (F3H; Unigene37233), and CYP92A1/flavonoid 3′-monooxygenase (F3’H; Unigene33426) genes were downregulated in both AA and FF5A in comparison with FF (Fig. 5A). *CHS* and *DFR* were the most upregulated genes in AA and FF5A (the log2 fold increase ranged from 6.2 to 8.31) relative to the FF genotype (Figs. 5A,C), whereas *CYP81D1* was the most downregulated one in AA and FF5A (−4.61 and −4.86 log2 fold decrease, respectively) relative to the FF genotype. Furthermore, we carried out a Pcc between AA and MALs based on a normalized gene expression fold change (Fig. 5D). Similar to the metabolomic correlation result, a positive correlation was observed between AA-FF5A (*r* = 0.50) and AA-FF7A (*r* = 0.47), whereas AA-FF2A and AA-FF4A displayed a weak positive correlation (*r* = 0.13 and 0.16, respectively).

### Phylogenetic analysis

**Figure 6:**
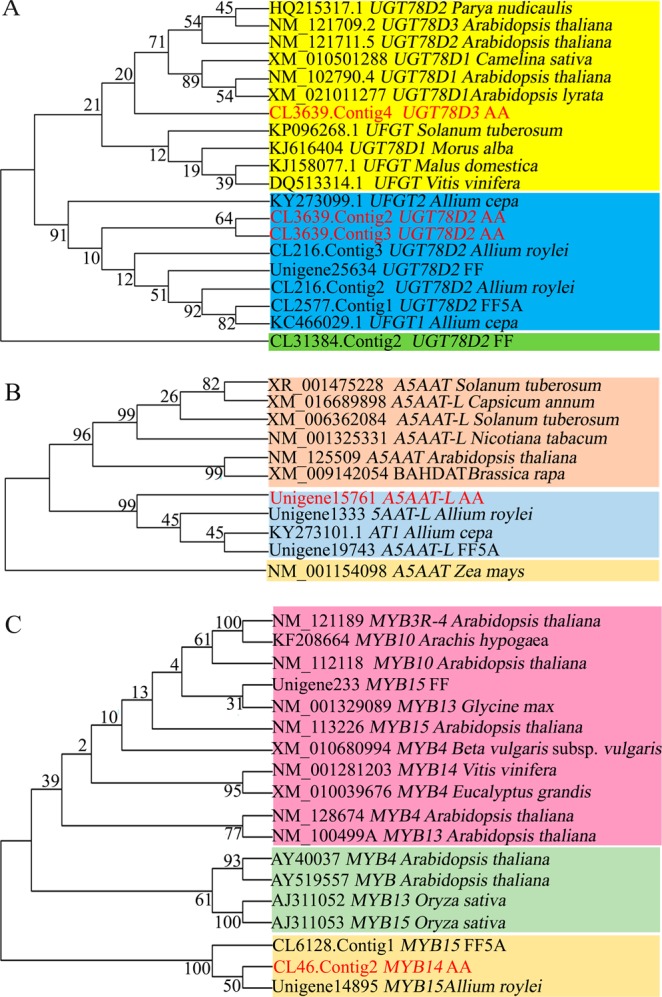
Neighbor-joining tree built from the number of differentially expressed genes in the *Allium* cepa Aggregatum group (AA) and *A*. *fistulosum* with extra chromosome 5A from the shallot. (**A**) *UDP-glucose flavonoid-3-O-glucosyltransferase* (*UGT78D2* and *UGT73D3*), (**B**) *aromatic acyltransferase-like* (*A5AAT-L*), and (**C**) *MYB14* transcriptome sequences obtained from our *Allium* TDB and their orthologs in different plant species derived from the National Center for Biotechnology Information (NCBI). A neighbor-joining tree with bootstrap values (1000 replicates) at all nodes was generated using MEGA 6.

Phylogenetic analysis was carried out using transcriptome-derived sequences of upregulated genes (*UGT78D2*, *UGT78D3*, *A5AAT/5A*, and *MYB14*) in both AA and FF5A, and their orthologs from different plant species were obtained from the NCBI (Figs. 6A–C). We used the neighbor-joining approach with 1000 bootstrap replicates to reconstruct phylogenetic trees, and bootstrap values are shown at all nodes (Figs. 6A–C). The phylogenetic tree showed high sequence similarity (identity 99%, E-value 0.0) between our UGT78D2 transcript (CL3639.Contig2 and CL3639.Contig3) and the onion *UFGT2* (accession KY273099.1) gene (Fig. 6A), whereas the UGT78D3 transcript (CL3639.Contig4) exhibited high sequence similarity with the *Arabidopsis thaliana UGT78D1* and *Solanum tuberosum UFGT* genes (accessions XM_021011277 and KP096268.1, respectively). Likewise, high sequence similarity (identity 98%, E-value 0.0) was detected between the A5AAT-L/5AT transcript (Unigene15761) and the onion *Acyltransferase1* (*AT1*; accession KY273101) gene (Fig. 6B). On the other hand, our transcript encoded by the MYB14 (CL46.Contig2) and *A*. *roylei* MYB15 genes were clustered separately from other Sg4 R2R3-MYBs derived from different plant species (Fig. 6C).

## Discussion

Onion bulb color is the result of the interaction of *C*, *I*, *G*, *L*, and *R* loci, among which one functional allele for the *C* locus and recessive alleles for the *I* locus are required for onion bulb color development, whereas other loci have modifying effects^2,25^. Onion bulb color ranged between white and red because of the variation in the production of different flavonoid compounds^2,26,27^. Flavonoid compounds can be divided into different flavonoid classes, with an estimated 10,000 different members, depending on the modification of the C-ring^28^. The flavonoid pathway is one of the well-characterized secondary metabolite pathways in model plants, and the transcriptional factors (TFs) are the key point for the regulation of flavonoid biosynthesis^2^. However, the several stages of flavonoid biosynthesis, especially *in vivo*, substrate specificity of glucosyltransferase, and acyltransferase in several *Allium* species, including shallots, require more elucidation. Additionally, little is known about flavonoid TFs in shallots. In this context, an integrated transcriptome and widely targeted metabolome analysis of MALs was carried out to investigate the genetic effect of the shallot (AA) genome on the *A*. *fistulosum* (FF) metabolome and transcriptome, with particular focus on the flavonoid biosynthetic pathway.

Bulb extracts were subjected to widely targeted metabolome analysis using LC-QqQ-MS, which resulted in the detection of 123 metabolite intensities (Supplementary Table S5). The trend in metabolic profiles of FF, AA, and MALs was compared using PCA analysis (Figs. 1A,B). PCA plots indicate a significant difference in the metabolic profiles between AA and FF (Figs. 1A–C). Amino acids, nucleic acids, and organic acids were characteristic for FF and FF2A, whereas di- and trisaccharides, including melibiose, sucrose, and 1F-fructosylsucrose, and several quercetin glucosides were characteristic for AA and other MALs (Figs. 1A–C). The accumulation of polysaccharides in shallots was recently reported in a comparative study between different shallot landraces and bulb onions^29^. The authors suggested that the accumulation of polysaccharides in the shallots, in comparison with bulb onions, contributed to the shallots’ adaptation to climatic conditions in subtropical regions^29^. Likewise, our recent study using comparative metabolome profiling of a shallot double haploid (DHA), onion double haploid (DHC), and F_1_ hybrid demonstrated a significant accumulation of carbohydrate, flavonoid, and phospholipid in the DHA and F_1_ hybrid in comparison with DHC, reflecting the adaptability of the two lines to abiotic stress conditions^9^. The heatmap hierarchical clustering of the metabolite fold change using the FF genotype as a control provides a further clear discrimination between AA and MALs (Fig. 2). AA and FF5A were clustered together with a significant accumulation of quercetin glycosides (quercetin-4′-glucoside, quercetin 3-galactoside, and quercetin-3,4′-diglucoside), flavone glycoside (luteolin-4′-O-glucoside) as well as anthocyanin glycosides (petunidin 3-O-glucoside) in comparison with other MALs (Fig. 2 and Supplementary Fig. S2). This result clearly demonstrates the genetic effect of a single addition chromosome 5A from the shallot on the *A*. *fistulosum* flavonoid pool. The addition of chromosome 5A from shallots induced the accumulation of flavonoids in FF, which is an important agronomic trait for both plant stress tolerance and human health. Previous reports demonstrated that shallots exhibited higher flavonoid levels than the bulb onion and Japanese bunching onion, which could be a metabolic phenotype characteristic that enables shallots to sustain the abiotic and biotic stress conditions in subtropical regions^9,16,29,30^. Sixteen flavonol compounds have been reported previously in onions and shallots, including the aglycone and glycosylated products of quercetin, kaempferol, and isorhamnetin^17,31,32^. Quercetin glucosides are the major flavonols present in onions and shallots and are mainly present in the form of quercetin, quercetin-4′-glucoside, and quercetin-3,4′-diglucoside^17,29,33^. However, the presence of flavone and anthocyanin glycosides, such as luteolin-4′-O-glucoside and petunidin 3-O-glucoside, respectively has not been reported in shallots.

**Figure 7:**
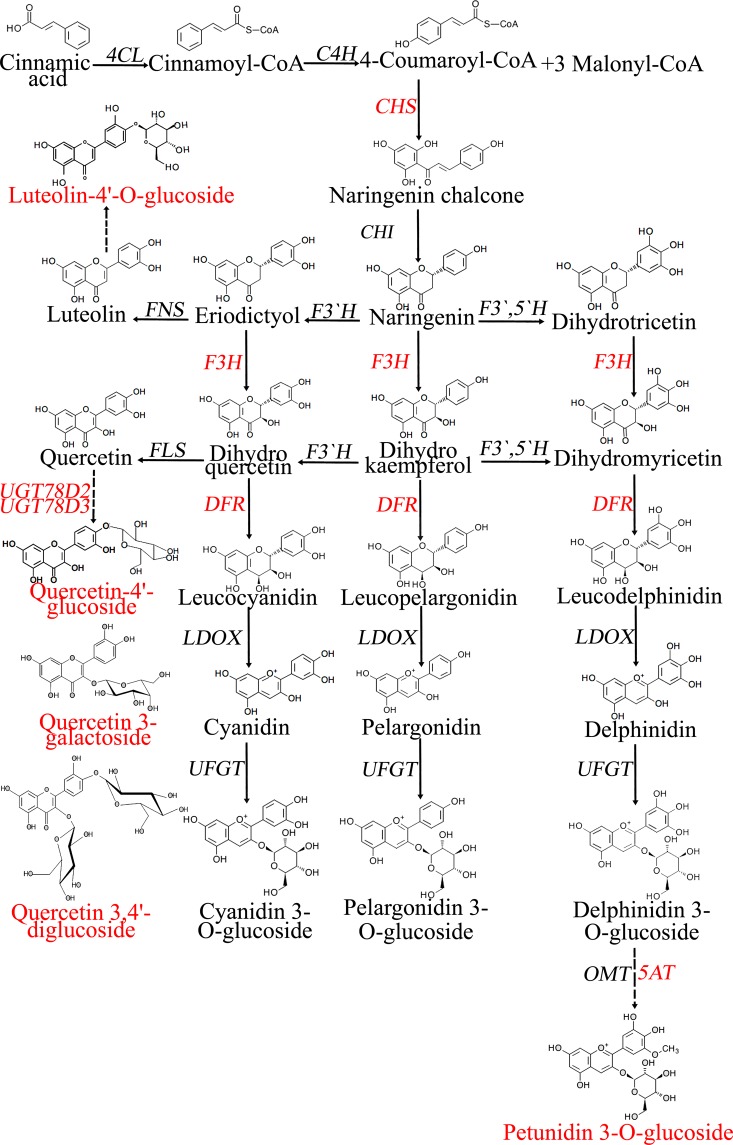
Schematic representation of the flavonoid biosynthesis pathway depicted from the Kyoto Encyclopedia of Genes and Genomes. Metabolites with significant accumulation in the *Allium cepa* Aggregatum group (AA) and *A*. *fistulosum* with extra chromosome 5A from the shallot (FF5A) are highlighted in red. Constitutively upregulated genes in AA and FF5A are also highlighted in red. *4-Coumarate–CoA ligase*, *4CL*; *trans-cinnamate 4-hydroylase*, *C4H*; *chalcone synthase*, *CHS*; *chalcone isomerase*, *CHI*; *flavanone 3-hydroxylase*, *F3H*; *dihydroflavonol 4-reductase*, *DFR*; *UDP-glucose flavonoid-3-O-glucosyltransferase*, *UFGT*; *5-aromatic acyltransferase*, *AT*; *O-methyltransferase*, *OMT*.

The accumulation of flavonoids in FF5A could result from the localization of flavonoid structural or regulatory genes on the shallot chromosome 5A. Therefore, we carried out transcriptome analyses using AA and MALs, with FF as a control (Fig. 5). The transcriptome analyses revealed that 13 unigenes were upregulated in both AA and FF5A, whereas eight unigenes were downregulated (Fig. 5B). In addition, the transcriptome analysis showed a positive correlation between AA and FF5A (*r* = 0.54), similar to the metabolomic data (Figs 4C and 5D), indicating a similar tendency in the metabolomic and transcriptomic profiles between AA and FF5A. The transcripts encoded by the flavonoid biosynthesis (*CHS*, *F3H*, and *DFR*), glycosylation (*UGT78D2* and *UGT73D3*), acylation (*A5AAT-L/5AT*), transporter (*PDR-L ABC*), and regulatory (*MYB14*) genes exhibited higher expression levels in both AA and FF5A than in FF (Figs. 5A,C and Supplementary Table S9). The expression of *CHS*, which encodes the enzyme for the first step in flavonoid biosynthesis, has been reported to be regulated by the *C* locus in onions^14^. The CHS enzyme plays an important role in providing chalcone scaffolds for the production of intermediate and final products of the flavonoid pathway (Fig. 7). DFR is a main enzyme in the anthocyanin biosynthesis that controls the stereospecific reduction of the 4-keto group of dihydroflavonols to the corresponding leucoanthocyanidins (Fig. 7), and a mutation within the *R* locus, which contains the *DFR* gene, is responsible for the variation between red and yellow onion cultivars^34^.

Although the central pathway for flavonoid biosynthesis is conserved in plants, a group of transferase enzymes, such as glycosyltransferase, acyltransferase, and methyltransferase, modify the flavonoid skeleton (Fig. 7), leading to the different flavonoid compounds within different plant species^35^. The glycosylation of flavonoids increases their solubility and stability in plants and affects their physiological activities and subcellular localization; therefore, the glycosylation represents an extremely important regulation point in the flavonoid homeostasis^35–37^. In the present study, several glycosyltransferase-related genes were upregulated in the shallot and/or FF5A, including *UGT72E3*, *UGT73B1*, *UGT73B5*, *UGT73C5*, *UGT78D2*, *UGT78D3*, and *UGT85A5* (Supplementary Table S9). Among these, *UGT78D2* and *UGT78D3* were upregulated in both AA and FF5A (Figs. 5A,C). UGT78D2 and UGT78D3 enzymes have been previously identified in *Arabidopsis* as family 1 glycosyltransferase, which utilizes UDP-sugars as sugar donors for flavonoid glycosylation^34^. UGT78D2 catalyzes the glycosylation of both flavonols and anthocyanins at the C-3 position, and the *Arabidopsis ugt78d2* mutant exhibited a low anthocyanin level and lacks 3-O-glucosylated quercetin as compared with wild-type plants^34,38^. Our transcripts (CL3639.Contig2 and CL3639.Contig3) encoded by the *UGT78D2* gene exhibited high sequence similarity with the onion *UFGT2* gene (Fig. 6A). A comparative expression of *UFGT2* in different onion cultivars demonstrated that *UFGT2* was highly expressed in the red onion cultivar, whereas a low expression level was detected in yellow and white onion cultivars^39^. UGT78D3 is a flavonol arabinosyltransferase enzyme that uses UDP-arabinose as a sugar donor and quercetin as a sugar acceptor, and the *Arabidopsis ugt78d3* mutant exhibited a lower accumulation of flavonol 3-O-arabinosides than did wild-type plants^40^. However, the correlation analysis demonstrated that UGT78D3 was weakly correlated with the flavonoid biosynthesis genes in comparison with UGT78D2^40^. In general, our data and previous results indicated that 3-O-glycosylations by UGT78D2 and UGT78D3 may play an important role in the flavonoid accumulation of the shallot (AA) through chromosome 5A.

Similar to glycosylation, the acylation of oxygen and nitrogen containing anthocyanins to produce esters and amides, respectively, by acyltransferase enzymes influences the stability of flavonoids at a neutral pH^41^. In addition, the flavonoid acylation plays a key role in flavonoid storage and transport into the vacuole^42^. In the present study, both AA and FF5A exhibited upregulation in the transcript (Unigene15761) encoded by the *A5AAT-L/5AT* gene (Figs. 5A,C). The phylogenetic analysis indicated that our transcriptome sequence of *A5AAT-L/5AT* exhibited high sequence similarity with the onion *AT1* and *A*. *roylei A5AAT-L* genes (Fig. 6B). A5AAT-L/5AT belongs to the BAHD acyltransferase family, which catalyzes the transfer of the coumaroyl or caffeoyl moiety to the 5-glucose of anthocyanidin 3,5-diglucosides in different plant species^41^. However, the molecular characterization of A5AAT/5AT in onions and shallots remains elusive. The aromatic acylation of anthocyanin in the grapevine (*Vitis vinifera*) shifts its color toward dark reddish blue or purple, and the acylated anthocyanins comprise more than 60% of the total anthocyanin contents in various *V*. *vinifera* cultivars^43^. Acylation is not only important for flavonoid color stability, but it also is essential for flavonoid transport in the grapevine. For example, antho-multidrug and toxic extrusion [antho-MATE1 (AM1)] and AM3 proteins mediate specifically acylated anthocyanin transport but could not transport cyanidin 3-O-glucoside or malvidin 3-O-glucoside, suggesting that the acyl conjugation was essential for the uptake^42^. Similar to many secondary metabolites, flavonoids are synthesized in the cytosol and transported into the vacuole to prevent cellular damage, where MATE and ATP-binding cassette (ABC) proteins are involved^44,45^. PDR-type ABC transporters have been reported in secretion of the flavonoid glycoside genistein from soybean roots^46^, and a multidrug resistance–associated protein (MRP), ZmMrp3, is required for anthocyanin transport in *Zea mays*^47^. However, the transporter system of flavonoids in onions and shallots remains unknown. In the present study, we identified a transcript encoded by the *PDR-L ABC* transporter gene that exhibited upregulation in both AA and FF5A in comparison with FF (Figs. 5A,C). The characterization of its *in vivo* role would supply more evidence of a relationship between the transport mechanism and the flavonoid composition in onions and shallots.

Transcriptional activation complex ‘MBW,’ consisting of R2R3-MYB and basic helix-loop-helix (bHLH) TFs, and a WD-repeat protein are thought to be the key regulatory points in determining the spatial and temporal accumulation of flavonoids in plants^2,27^. In Asiatic lilies, R2R3-MYB (LhMYB6) was able to activate the promoters of the *CHSa* and *DFR* genes and positively regulate anthocyanin biosynthesis only when LhMYB6 form a protein-protein heterodimer interaction with bHLH2^48^. Similarly Schwinn *et al*. (2016) demonstrated that MYB1 serves as a positive regulator of anthocyanin production in onions by regulating *CHS* and *DFR* expression^2^. In the present study, *MYB14*, *MYB15*, and *MYB111* TFs were upregulated, whereas *MYB33*, *MYB60*, *ENHANCER OF GLABRA3* (*EGL3*/*bHLH*) TFs, and the *AN11/WD40-*repeat protein were downregulated in AA and/or FF5A relative to FF (Fig. 5A and Supplementary Table S9). *MYB14* exhibited upregulation in both AA and FF5A (Figs. 5A,C). A recent study^49^ demonstrated the involvement of *MYB14* and *MYB15* in the transcriptional regulation of stilbene synthases (STSs), which are key enzymes for stilbene biosynthesis, in grapevines. Stilbene biosynthesis is a side branch of the flavonoid pathway through STSs, which compete with CHS for *p*-coumaroyl-CoA and cinnamoyl-CoA substrates for the synthesis of resveratrol derivatives^49^. A rapid and strong upregulation in *Pinus taeda PtMYB14* in response to mechanical wounding, jasmonic acid treatment, or cold stress has been reported^50^. In addition, *P*. *taeda PtMYB14* overexpression lines accumulated large amounts of anthocyanin O-glucosides, including cyanidin 3-O-glucoside, delphinidin 3-O-glucoside, peonidin 3-O-glucoside, petunidin 3-O-glucoside, and malvidin 3-O-glucoside, suggesting that PtMYB14 is involved in the *P*. *taeda* defense response through flavonoid accumulation^50^. In the future, characterization of the MYB14 role in shallot flavonoid biosynthesis and its target gene(s) would provide further clarification regarding the TF regulation of flavonoid biosynthesis in shallots and onions.

In conclusion, this study provides a comprehensive evaluation of the genetic effect of shallot chromosome 5A on the *A*. *fistulosum* metabolome. Integrated widely targeted metabolome and transcriptome analyses enabled us to identify several quercetin, flavone and anthocyanin glucosides in the bulb extract of the FF genome with an extra chromosome 5A from the shallot. The transcriptome data showed an upregulation of biosynthesis (*CHS*, *F3H*, and *DFR*), glycosylation (*UGT78D2* and *UGT78D3*), acylation (*A5AAt/5AT*), transport (*PDR-L ABC*), and regulatory (*MYB14*) genes in both AA and FF5A relative to FF (Fig. 5A). The upregulation of these genes in AA and FF5A in comparison with FF could be the switch key for flavonoid accumulation in these genotypes. The *in vivo* characterization of these genes using transgenic lines will provide further validation of their role in shallot and onion flavonoid biosynthesis.

## Methods

### Plant materials

*A*. *fistulosum* (FF), shallots (AA), and eight different MALs (FF1A, FF2A, FF3A, FF4A, FF5A, FF6A, FF7A, and FF8A)^10^ were grown in clay pots filled with sand. Each pot contained one plant, and all of the plant genotypes were vegetatively propagated under the same conditions at the Yamaguchi University greenhouse (34°N, 131°E) with a 25 ± 2 °C average temperature, 78% relative humidity, and 10 h daylight length. Water and fertilizers were applied equally for all genotypes on a weekly basis. Plants were collected from each line separately in biological replicates (n = 3). After cleaning, bulb tissues [3 replicates × 10 genotypes (FF, AA, and eight MALs)] were cut separately and immediately frozen in liquid nitrogen for RNA extraction and metabolome analysis.

### Widely targeted metabolomics of FF, AA, and MALs

Bulb tissues [3 replicates × 10 genotypes (FF, AA, and eight MALs)] were freeze-dried using a rotary evaporator (BUCHI, Rotavapor R-3) coupled with a vacuum pump (BUCHI, v-700) under reduced pressure at 10 °C ± 1. The sample preparation process was performed automatically by a liquid handling system (Microlab Star Plus, Hamilton) as described by Sawada *et al*. (2017)^51^. Briefly, 4 mg dry weight of bulb tissues was accurately weighted and transferred into a 2 ml tube with a 5 mm Zirconia bead. The metabolites were extracted using a proportional volume of 4 mg ml^−1^ extraction solvent (80% methanol, 0.1% formic acid, 16.8 nmol L^−1^ lidocaine, and 105 nmol L^−1^ 10-camphorsulfonic acid as internal standards) using a multi-bead shocker (Shake Master NEO, Bio Medical Science) at 1000 rpm for 5 min. After centrifugation, the extracts were diluted to 40 µg ml^−1^ using an extraction solvent. Then 250 µL of the extract was transferred to a 96-well plate, dried, redissolved in 250 µL of ultra-pure water, and filtered using Whatman^®^ UNIFILTER^®^ plates 384 (GE Healthcare). One microliter of the solution extract at a final concentration of 40 µg ml^−1^ was subjected to widely targeted metabolomics using LC-QqQ-MS (UPLC-TQS, Waters). A KEGG ID and name, as well as our internal standard ID, were assigned to each metabolite. For detailed information, see Supplementary Tables S1–S4.

### Metabolomic data analysis

The metabolomic data matrix of 499 metabolite intensities was obtained via LC-QqQ-MS analysis (Supplementary Table S1). The metabolomic data were analyzed using the statistical software R (version 3.2.2, http://www.R-project.org/). After the missing values were set to 20, the signal intensities of each experimental group were averaged in individual metabolites. The metabolites with a S/N (defined as ratio of average signal intensity to that of the extraction solvent control) < 5 in all experimental groups were removed. In addition, the metabolites with RSD > 0.30 in all experimental groups were removed, leaving 123 metabolites for further analysis. The intensities of the 123 metabolites were divided by those of the internal standards, and the resulting data matrix was used for comparative analysis (Supplementary Table S5). Initially, all 123 metabolites were subjected to pathway analysis using the MetaboAnalysis 4.0 database (http://www.metaboanalyst.ca/). Then a multivariate analysis was performed to evaluate the general trend in the metabolomic data in the different genotypes. The significant (*P* < 0.05) accumulation of metabolite intensities in each genotype was analyzed via *t*-test using FF genotypes as a control. A correlation analysis between AA and MALs was carried out using the Pcc in R v. 3.2.2. In addition, all of the significantly (*P* < 0.05) accumulated metabolites in both AA and FF5A, in comparison with FF, were used to generate a metabolite network. The metabolite network was developed based on the Pcc using Correlation Calculator 1.0.1, and the network was plotted using Cytoscape 3.3.0 based on *Arabidopsis* database with the following parameters: base edges on the Pcc, tooltip labels none, and range for edges from −1.0 to 1.0.

### Transcriptome analysis of FF, AA, and MALs

Total RNA was extracted using an RNeasy Plant Mini Kit (QIAGEN Sciences, Germantown, Maryland, USA). RNA quality was assessed using a BioSpec-nano (Shimadzu, Kyoto, Japan) and Agilent 2100 Bioanalyzer (Agilent Technologies, Palo Alto, CA, USA). The cDNA library was constructed using TruSeq™ RNA Sample Preparation Kit (Illumina, San Diego, CA, USA) in accordance with the manufacturer’s instructions. The cDNA library was prepared in accordance with Illumina’s protocol, and paired-end sequencing was performed using Illumina’s HiSeq 2500 platform. The raw quality reads including adapter sequence and unknown nucleotides more than 5% and low-quality nucleotides (QV < 10) more than 20% in length were respectively excluded. The clean reads were assembled into contigs by Trinity r20121005 with the parameters -seqType fq-min_contig_length 100_group_pairs_distance 250-path_reinforcement_distance 85-min_kmer_cov 2^52^. In each sample, the contigs were clustered by a TGI Clustering Tool (TGICL) v2.1^53^ with the parameters -l 40 -c 10-v 20 and further assembled into unigenes by Phrap 23.0^54^ with the parameters -repeatstringency 0.95 -minmatch 35 -minscore 35. We searched 56,161 unigene sequences against the International Rice Genome Sequencing Project (IRGSP 1.0; http://rgp.dna.affrc.go.jp/IRGSP/), the *Arabidopsis* Information Resource (TAIR10; http://www.arabidopsis.org), and the NCBI’s non-redundant (nr) protein database (http://www.ncbi.nlm.nih.gov) using the BLASTX^55^ program with an E-value cutoff of 1E-10. Based on the number of reads mapped onto the unigene sequences, the reads per kilobase per million mapped reads (RPKM) value of each gene was calculated using an in-house Perl script. We tested for differences between the normalized means of AA and MALs compared with FF as a control. Genes were classified as differentially expressed if they exhibited ≥2-fold changes and a false discovery rate (FDR) < 0.05 using EdgR. All of the transcriptome data, including gene expression, sequence data, and gene annotation, were deposited in our open access *Allium* TDB.

### Phylogenetic analysis of flavonoid glycosylation, acylation, and TF genes

The phylogenetic tree was developed by using the nucleotide sequences of glycosylation, acylation, and TF genes from FF, AA, and FF5A located in our *Allium* TDB, and their orthologs were obtained from the NCBI. The sequence alignments were constructed with a ClustalW package in BioEdit software. Then the phylogenetic tree was developed using the neighbor-joining method with bootstrap values (1000 replicates) using the MEGA 6.0 program.

## Supplementary information

Supplementary Figs.1–2

Supplementary Tables 1–9
