# Supplementary material for: Widely targeted metabolome and transcriptome landscapes of Allium fistulosum–A. cepa chromosome addition lines revealed a flavonoid hot spot on chromosome 5A
Source: Sci Rep. 2019 Mar 5;9:3541. doi: 10.1038/s41598-019-39856-1 (PMC6400954; doi:10.1038/s41598-019-39856-1)
Supplement: Supplementary file 1 — Supplementary Figs.1–2 [file 41598_2019_39856_MOESM1_ESM.pdf]

**Widely targeted metabolome and transcriptome landscapes of *Allium fistulosum–A. cepa* chromosome addition lines revealed a flavonoid hot spot on chromosome 5A**

Mostafa Abdelrahman,[1,2] Sho Hirata[3], Yuji Sawada,[4] Shusei Sato,[5] Hideki Hirakawa,[6] Yoko Mine,[7] Keisuke Tanaka,[8] Masayoshi shigyo[9,*]

[1]Botany Department, Faculty of Science, Aswan University, Aswan 81528, Egypt

[2]Arid Land Research Center, Tottori University, Tottori 680-0001, Japan

[3]Nakahara Seed Product, Fukuka 812-0893, Japan

[4]RIKEN Center for Sustainable Resource Science, Yokohama, Kanagawa 230-0045, Japan

[5]Graduate School of Life Sciences, Tohoku University, Aoba-ku, Sendai 980-8577, Japan

[6]Kazusa DNA, Research Institute, Kisarazu, Chiba 292-0818, Japan

[7]Department of Agriculture, Faculty of Agriculture, Tokyo University of Agriculture, Tokyo 113-8654, Japan

[8]NODAI Genome Research Center, Tokyo University of Agriculture, Tokyo 156-8502, Japan

[9]Laboratory of Vegetable Crop Science, College of Agriculture, Graduate School of Sciences and Technology for Innovation, Yamaguchi 753-8512, Japan

* Correspondence and requests for materials should be addressed to MS (shigyo@yamaguchi-u.ac.jp).

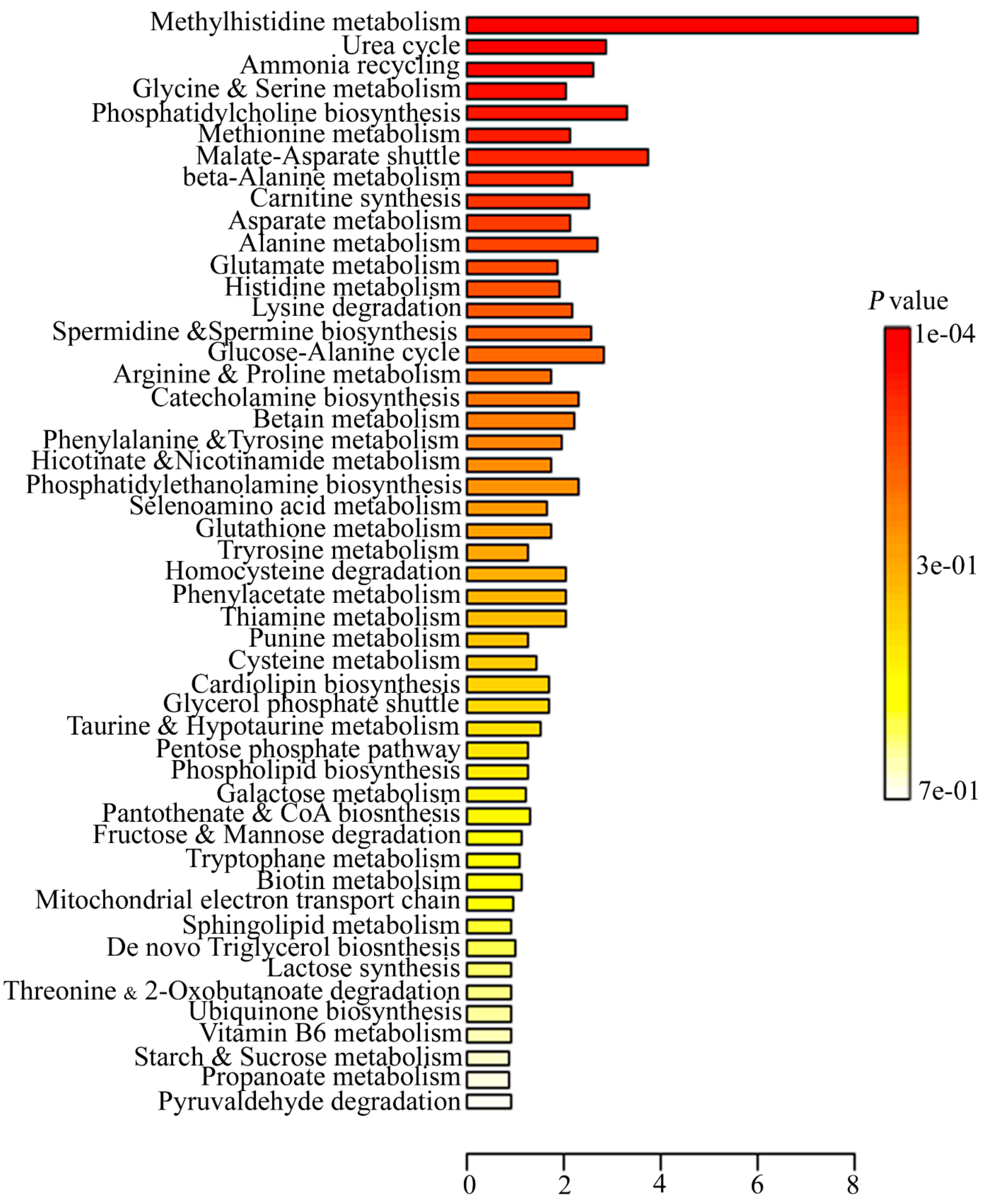


**Supplementary Figure S1.** Enrichment pathway analysis of all 123 metabolites using MetaboAnalyst 4.0.

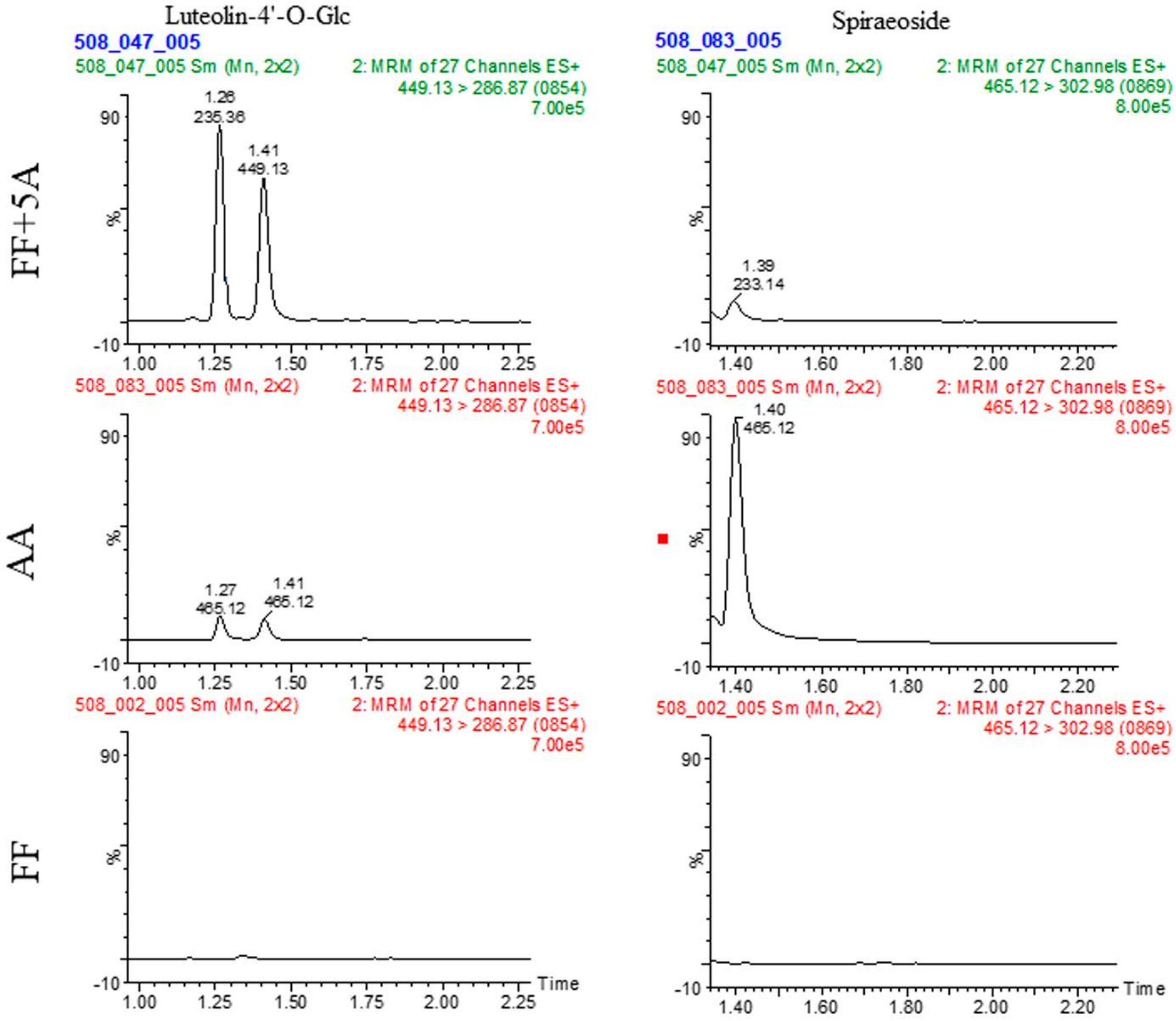


**Supplementary Figure S2.** Ultra-high-performance liquid chromatography–photo diode array (UPLC-PDA) chromatograms with assigned mass spectra for luteolin-4’-O-glucoside and spiraeoside/quercetin-3,4’-diglucoside in the *Allium cepa* L. Aggregatum group (AA), *A. fistulosum* (FF), and *A. fistulosum* with extra chromosome 5A from the shallot (FF5A). In the y-axis, ion count intensities were adjusted at 7 × 10^5 for luteolin-4'-O-glc and 8 × 10^5 for spiraeoside/quercetin-3,4’-diglucoside. The x-axis represents the retention time.
